# lncRNA MALAT1 Accelerates Skeletal Muscle Cell Apoptosis and Inflammatory Response in Sepsis by Decreasing *BRCA1* Expression by Recruiting EZH2

**DOI:** 10.1016/j.omtn.2019.10.028

**Published:** 2019-11-02

**Authors:** Hui Yong, Gangming Wu, Jingyuan Chen, Xueru Liu, Yiping Bai, Ni Tang, Li Liu, Jicheng Wei

**Affiliations:** 1 Department of Anesthesiology, The First Affiliated Hospital of Southwest Medical University, Luzhou 646000, P.R. China; 2 Department of Anesthesiology, The First Affiliated Hospital of Chongqing Medical University, Chongqing 400016, P.R. China

**Keywords:** long non-coding RNA MALAT1, sepsis, EZH2, BRCA1, apoptosis, inflammatory response, skeletal muscle cells

## Abstract

Sepsis is a serious and elusive syndrome caused by infection, which is accompanied by a high mortality worldwide. Recent evidence has documented the regulatory role of long non-coding RNA (lncRNA) metastasis-associated lung adenocarcinoma transcript 1 (MALAT1) during the inflammatory process, the effects of which in the development of sepsis have become the focus of the current study. An *in vivo* mouse model and *in vitro* cell model of sepsis induced by lipopolysaccharide (LPS) were developed. High expression of lncRNA MALAT1 along with low expression of breast cancer susceptibility gene 1 (*BRCA1*) were identified in septic mice and human skeletal muscle cells of sepsis. Then, lncRNA MALAT1 expression was altered *in vivo* and *in vitro* to examine serum levels of inflammatory factors, as well as skeletal muscle cell apoptosis. lncRNA MALAT1 was noted to regulate the expression and export from the nucleus of *BRCA1* by recruiting zeste homolog 2 (EZH2) in skeletal muscle cells of sepsis. Silencing lncRNA MALAT1 resulted in reduced serum levels of interleukin (IL)-6, IL-8, and tumor necrosis factor alpha (TNF-α), neutrophil migration, skeletal muscle cell apoptosis, and AKT-1 phosphorylation. Taken together, lncRNA MALAT1 interacting with EZH2 stimulated AKT-1 phosphorylation and decreased *BRCA1* expression, consequently aggravating the progression of sepsis, highlighting a promising therapeutic option for sepsis.

## Introduction

Sepsis is a clinical syndrome that is caused by the dysregulated inflammatory response to infection.^1^ The incidence of sepsis is reported to be increasing, and the absolute number of sepsis-related mortality cases is considerably high in China.^2^ Sepsis frequently occurs in elderly people, the most severe form of which can lead to multiple organ dysfunction that will cause critical illness accompanied by severe immune dysfunction and catabolism.^3^ The early diagnosis and stratification of the severity of sepsis is very important, which can improve the outcome by using timely and specific treatment.^4^ However, the diagnosis and severity evaluation of sepsis are complicated by the highly variable and non-specific nature of its signs and symptoms.^5^

In recent years, long non-coding RNAs (lncRNAs) have functioned as novel gene regulators as well as prognostic markers in sepsis.^6^ The metastasis-associated lung adenocarcinoma transcript 1 (MALAT1), also known as nuclear-enriched transcript 2, is a lncRNA that has been identified to be involved in several human tumors.^7^ For example, overexpression of lncRNA MALAT1 confers an oncogenic function in renal cell carcinoma.^8^ It has also been reported that lncRNA MALAT1 can regulate sepsis-induced cardiac inflammation and dysfunction.^9^ However, the expression of lncRNA MALAT1 and its significance in sepsis remain largely unknown. lncRNA MALAT1 was able to interact with enhancer of zeste homolog 2 (EZH2), which is a histone methyltransferase.^8^ A previous study has demonstrated that EZH2 can inhibit breast cancer susceptibility gene 1 (*BRCA1*) in breast cancer.^10^ *BRCA1*, along with *BRCA2*, is a breast cancer susceptibility gene and a well-known tumor suppressor gene that displays an autosomal dominant pattern of inheritance accompanied by high penetrance.^11^ Gene therapy with *BRCA1* has been shown to decrease the systemic inflammatory response, multiple organ failure, and mortality, and consequently to improve survival in sepsis.^12^ Previous evidence has indicated that the protein kinase B/mammalian target of rapamycin (AKT/mTOR) signaling pathway is implicated in the improvement of brain dysfunction by exogenous recombinant human erythropoietin through reducing neuronal apoptosis in sepsis.^13^ Hence, the current study was designed with the aim of investigating the potential role of lncRNA MALAT1 in the initiation and development of sepsis, and we have demonstrated that lncRNA MALAT1 functions through the EZH2/*BRCA1*/AKT-1 axis to affect the progression of sepsis.

## Results

### Upregulated lncRNA MALAT1 and Downregulated *BRCA1* Are Found in Skeletal Muscle Tissues of Septic Mice

**Figure 1:**
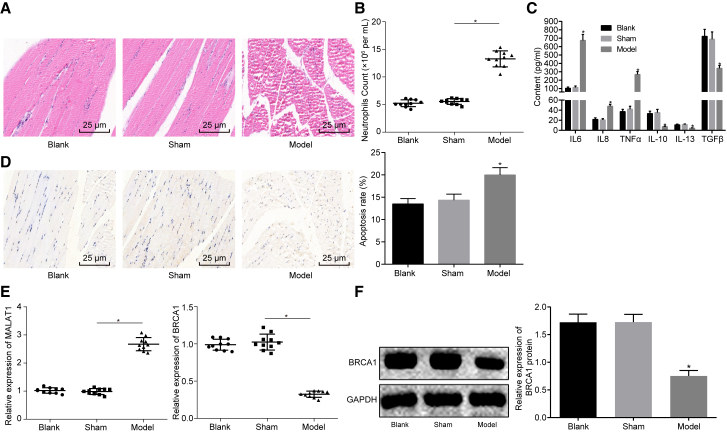
lncRNA MALAT1 Is Upregulated and *BRCA1* Is Downregulated in Skeletal Muscle Tissues of Septic Mice (A) The degree of skeletal muscle tissue injury in septic mice observed by H&E staining (×400). (B) The number of neutrophils in peripheral blood in septic mice measured by flow cytometry. (C) The serum levels of IL-6, TNF-α, IL-8, IL-10, TGF-β, and IL-13 measured by ELISA. (D) The apoptosis of skeletal muscle cells measured by TUNEL staining (×400). (E) The expression of MALAT1 and *BRCA1* in skeletal muscle tissues determined by qRT-PCR. (F) The expression of *BRCA1* normalized to GAPDH in skeletal muscle tissues determined by western blot analysis. *p < 0.05 versus the blank group or the sham group. The statistical values were measurement data, expressed as mean ± SD, and were analyzed with one-way ANOVA, followed by Tukey’s *post hoc* test. N = 10. The experiment was repeated three times independently.

Previously, accumulating evidence has shown that lncRNAs are involved in the pathogenesis of sepsis.^14^, ^15^, ^16^ Specifically, it has been suggested that lncRNA MALAT1 is upregulated in a rat model of sepsis, which regulates myocardial inflammation and induces myocardial dysfunction.^9^^,^^17^ Additionally, adenovirus-mediated *BRCA1* can alleviate the recruitment of neutrophils and the expression of inflammatory factors in septic mice, which exerts therapeutic effects on septic mice.^12^ Sepsis can reduce a muscle’s capacity for force production and skeletal muscle mobility, therefore inducing muscle atrophy and serious skeletal muscle injury. However, research on skeletal muscle injury in sepsis is scant. In our study, in order to explore the regulatory mechanism of skeletal muscle injury in sepsis, we constructed a mouse model of sepsis by lipopolysaccharide (LPS) induction. Then, H&E staining was performed to detect the degree of skeletal muscle injury, which showed that the diameter and area of muscle fibers in the septic mice increased significantly (Figure 1A). Next, flow cytometry was carried out to detect the number of neutrophils in peripheral blood of septic mice, which revealed that the number of neutrophils in the sepsis group was increased significantly (Figure 1B). Subsequently, ELISA was performed to determine the levels of inflammatory factors, including interleukin (IL)-6, tumor necrosis factor alpha (TNF-α), IL-8, IL-10, transforming growth factor β (TGF-β), and IL-13 in peripheral blood. It was suggested that the serum levels of IL-6, IL-8, and TNF-α were significantly higher whereas those of IL-10, IL-13, and TGF-β were significantly lower in the sepsis group than those in the sham group (Figure 1C). Terminal deoxynucleotidyl transferase (TdT)-mediated 2′-deoxyuridine 5′-triphosphate (dUTP) nick end labeling (TUNEL) staining was conducted in order to detect the apoptosis of skeletal muscle cells, which showed that the number of apoptotic cells increased significantly in the sepsis group (Figure 1D). All of the above results suggested that the sepsis model of mice was successfully constructed.

Then, the expression of lncRNA MALAT1 and *BRCA1* in skeletal muscle tissues of septic mice was detected by qRT-PCR. It was indicated that lncRNA MALAT1 was significantly upregulated and *BRCA1* was significantly downregulated in the sepsis group (Figure 1E). Western blot analysis also showed that the expression of *BRCA1* decreased in skeletal muscle tissues of septic mice (Figure 1F). Therefore, lncRNA MALAT1 was expressed at a high level and *BRCA1* was expressed at a low level in skeletal muscle tissues of septic mice.

### lncRNA MALAT1 Affects the Expression of *BRCA1* in Human Skeletal Muscle Cells of Sepsis

**Figure 2:**
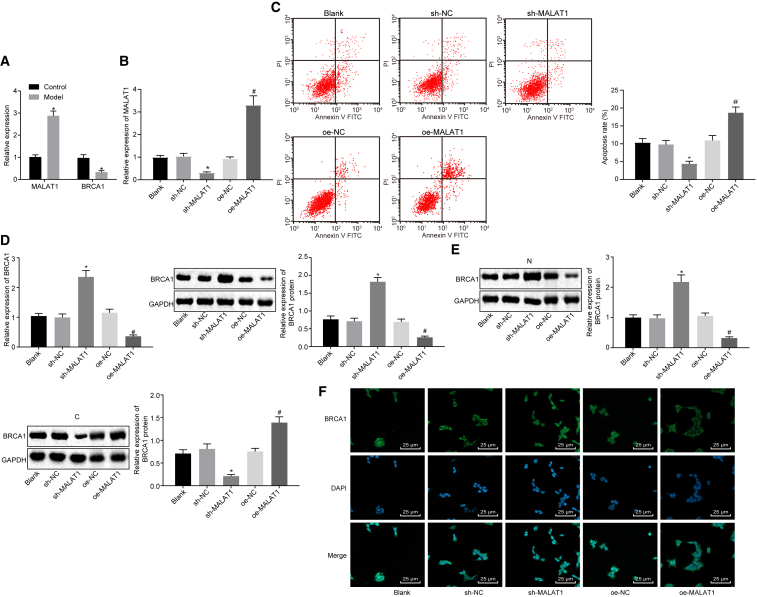
lncRNA MALAT1 Affects the Expression of *BRCA1* in Human Skeletal Muscle Cells (HSMKMC 3500) of Sepsis (A) The expression of lncRNA MALAT1 and *BRCA1* in normal skeletal muscle cells and septic skeletal muscle cells determined by qRT-PCR (the control group represents normal skeletal muscle cells; the sepsis group represents the LPS-induced skeletal muscle cell model of sepsis). (B) The silence efficiency of lncRNA MALAT1 in the skeletal muscle cell model of sepsis determined by qRT-PCR (blank, skeletal muscle cell model of sepsis; sh-NC, skeletal muscle cells in sepsis transfected with sh-NC; sh-MALAT1, skeletal muscle cells in sepsis transfected with sh-MALAT1). (C) The apoptosis of skeletal muscle cells measured by flow cytometry. (D) The expression of *BRCA1* and BRCA1 in skeletal muscle cells of sepsis determined by qRT-PCR and western blot analysis. (E) The expression of BRCA1 normalized to GAPDH in the nucleus and cytoplasm determined by western blot analysis. (F) The subcellular localization of BRCA1 in skeletal muscle cells of sepsis detected by an immunofluorescence assay (×400). *p < 0.05 versus the control, blank, or sh-NC group. The statistical values were measurement data, expressed as mean ± SD. The data between two groups were analyzed with an independent samples t test, and the data among multiple groups were analyzed using one-way ANOVA, followed by Tukey’s *post hoc* test. The experiment was repeated three times independently.

To further explore the potential relationship between lncRNA MALAT1 and *BRCA1*, we induced a sepsis model using human skeletal muscle cells (HSMKMC 3500) by LPS treatment. It was shown that the expression of lncRNA MALAT1 was upregulated and the expression of *BRCA1* was downregulated in HSMKMC 3500 cells after LPS treatment (Figure 2A). Then, lncRNA MALAT1 was overexpressed and silenced in HSMKMC 3500 cells, which was confirmed by qRT-PCR (Figure 2B). Then, we detected the apoptosis of cells after lncRNA MALAT1 was overexpressed and silenced by flow cytometry. It was demonstrated that lncRNA MALAT1 silencing remarkably decreased the number of apoptotic cells (Figure 2C). The expression of *BRCA1* in cells was determined by qRT-PCR and western blot analysis, which showed that the expression of *BRCA1* increased significantly after lncRNA MALAT1 silencing and decreased in response to overexpressed lncRNA MALAT1 (Figure 2D), indicating that lncRNA MALAT1 could affect the expression of *BRCA1*. To further explore the regulatory mechanism of lncRNA MALAT1 on *BRCA1*, we determined the expression of BRCA1 in the nucleus and cytoplasm, respectively, by western blot analysis. It was indicated that lncRNA MALAT1 silencing could promote the expression of BRCA1 in the nucleus and reduce the expression of BRCA1 in the cytoplasm (Figure 2E) while overexpressed lncRNA MALAT1 tended to induce opposite results. Moreover, the subcellular localization of *BRCA1* was then detected by an immunofluorescence assay, the results of which were consistent with western blot analysis (Figure 2F). These findings suggested that lncRNA MALAT1 regulated the expression and export from the nucleus of *BRCA1*.

### lncRNA MALAT1 Regulated Expression of *BRCA1* via Recruiting EZH2 in Human Skeletal Muscle Cells of Sepsis

**Figure 3:**
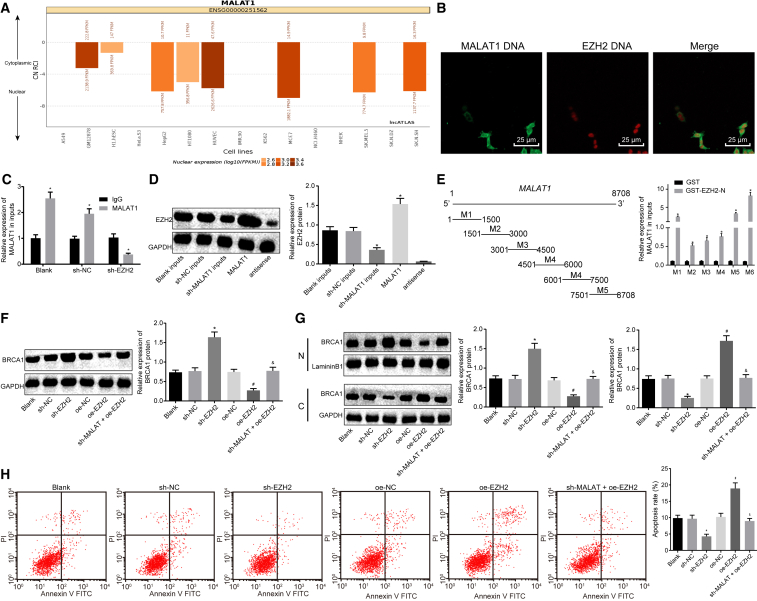
lncRNA MALAT1 Regulates Expression of *BRCA1* via Recruiting EZH2 in Skeletal Muscle Cells of Sepsis (A) The predicted subcellular location of lncRNA MALAT1 by the lncATLAS website. (B) The co-location of lncRNA MALAT1 and EZH2 in skeletal muscle cells of sepsis determined by RNA-FISH (original magnification, ×400). (C) The binding of lncRNA MALAT1 and EZH2 detected by RIP assay. *p < 0.05 versus the IgG group. (D) The binding of lncRNA MALAT1 and EZH2 confirmed by an RNA pull-down assay. (E) The binding of truncated lncRNA MALAT1 with EZH2 by RIP assay. *p < 0.05 versus the GST group. (F) The expression of BRCA1 normalized to GAPDH determined by western blot analysis. (G) The expression of BRCA1 in the nucleus and cytoplasm normalized to LamininB1 and GAPDH, respectively, determined by western blot analysis. (H) The apoptosis of cells measured by flow cytometry. *p < 0.05 versus the sh-NC group; ^#^p < 0.05 versus the oe-NC group; ^&^p < 0.05 versus the oe-EZH2 group. The statistical values were measurement data and are expressed as mean ± SD. The data between two groups were analyzed with an independent samples t test, and the data among multiple groups were analyzed using one-way ANOVA, followed by Tukey’s *post hoc* test. The experiment was repeated three times independently.

It is reported that lncRNA MALAT1 binds to EZH2 in castration-resistant prostate cancer.^18^ The prediction from the lncATLAS website (http://lncatlas.crg.eu) showed that lncRNA MALAT1 was mainly located in the nucleus (Figure 3A). Therefore, we detected the co-localized expression of lncRNA MALAT1 and EZH2 by an RNA fluorescence *in situ* hybridization (FISH) assay, which showed that both lncRNA MALAT1 and EZH2 were located in the nucleus (Figure 3B), indicating that lncRNA MALAT1 and EZH2 might also interact with each other in skeletal muscle cells. Furthermore, we detected the binding of lncRNA MALAT1 to EZH2 protein by a RNA-binding protein immunoprecipitation (RIP) assay, which showed that the enrichment of lncRNA MALAT1 in the blank and short hairpin RNA negative control (sh-NC) groups was significantly higher than that in the sh-EZH2 group (Figure 3C). Subsequently, an RNA pull-down assay and western blot analysis were carried out, the results of which suggested that the level of EZH2 pulled down in the blank and sh-NC groups was significantly higher than that in the sh-MALAT1 group (Figure 3D). Furthermore, the RIP assay indicated that the 7,500- to 8,700-bp region of lncRNA MALAT1 interacted directly with the N-terminal of EZH2 (Figure 3E). These results suggested that lncRNA MALAT1 can directly bind to EZH2 protein. Furthermore, we detected the expression of BRCA1 in sepsis skeletal muscle cells after the expression of EZH2 was altered by western blot analysis. It was shown that silencing EZH2 could promote the expression of BRCA1. Moreover, the expression of BRCA1 was inhibited by overexpressing EZH2, which could be restored by sh-MALAT1 (Figure 3F). The subcellular localization of *BRCA1* in sepsis cells in response to sh-EZH2 or overexpressed EZH2 (oe-EZH2) was assessed by western blot analysis. The results revealed that EZH2 silencing could promote the expression of BRCA1 in the nucleus and inhibit the expression of BRCA1 in the cytoplasm. Additionally, overexpression of EZH2 could inhibit the expression of BRCA1 in the nucleus and promote the expression of BRCA1 in the cytoplasm, which could be blocked by silencing lncRNA MALAT1 at the same time (Figure 3G). Flow cytometry was performed to detect the apoptosis of cells in different groups. Silencing EZH2 reduced the number of apoptotic cells, and overexpression of EZH2 increased the number of apoptotic cells, which could be reversed in response to sh-MALAT1 (Figure 3H). These results indicated that lncRNA MALAT1 could potentially regulate the expression as well as the subcellular localization of *BRCA1* and the apoptosis of sepsis skeletal muscle cells by recruiting EZH2.

### EZH2 Regulates *BRCA1* Expression Independent of Its Methyltransferase Activity

**Figure 4:**
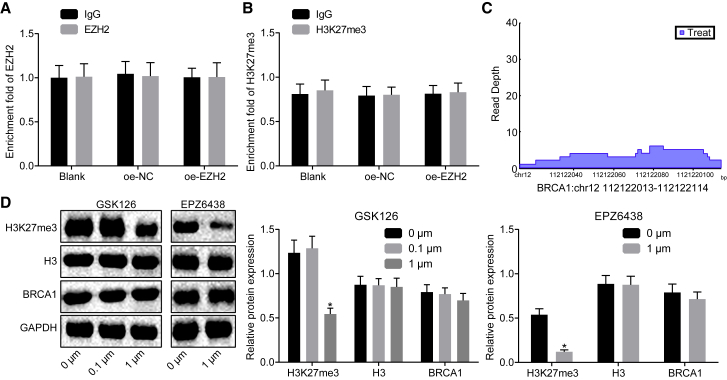
EZH2 Regulates *BRCA1* Expression Independent of Methyltransferase Activity (A) The enrichment of EZH2 in the promoter region of BRCA1 detected by ChIP. (B) The enrichment of H3K27me3 in the promoter region of *BRCA1* detected by ChIP. (C) The enrichment of EZH2 in the intron region of *BRCA1* analyzed by microarray-based analysis. (D) The expression of BRCA1 normalized to GAPDH in skeletal muscle cells treated with GSK126 and EPZ6438 (inhibitor of K3K27me3) determined by western blot analysis. The statistical values were measurement data and are expressed as mean ± SD; the data between two groups were analyzed with an independent samples t test. The experiment was repeated three times independently.

**A**ccumulating evidence has shown that EZH2 not only acts as a methyltransferase for epigenetic regulation, but it also serves as a non-epigenetic regulator.^19^, ^20^, ^21^ To further verify the mechanism of EZH2 regulating BRCA1, a chromatin immunoprecipitation (ChIP) assay was conducted to detect whether EZH2 was enriched in the promoter region of *BRCA1*. It was shown that EZH2 exhibited no enrichment in the promoter region of *BRCA1* in skeletal muscle cells overexpressing EZH2 (Figure 4A). Then, the enrichment of trimethylation modification on histone H3 lysine27 (H3K27me3) in the *BRCA1* promoter region was detected by a ChIP assay, which suggested that there emerged no enrichment of H3K27me3 in *BRCA1* promoter detected in skeletal muscle cells overexpressing EZH2 (Figure 4B). Microarray-based analysis revealed that EZH2 was enriched in the intron region of *BRCA1* (Figure 4C). qRT-PCR and western blot analysis were carried out in order to detect the expression of *BRCA1* in skeletal muscle cells treated with GSK126 and EPZ6438 (inhibitor of EZH2 methyltransferase activity), which showed that treatment of GSK126 and EPZ6438 had no significant effect on the expression of *BRCA1* (Figure 4D). These results suggested that EZH2 regulated *BRCA1* expression independent of its methyltransferase activity.

### lncRNA MALAT1 Regulates *BRCA1* Expression through Promoting the Phosphorylation of AKT-1 via Recruiting EZH2 in Human Skeletal Muscle Cells of Sepsis

**Figure 5:**
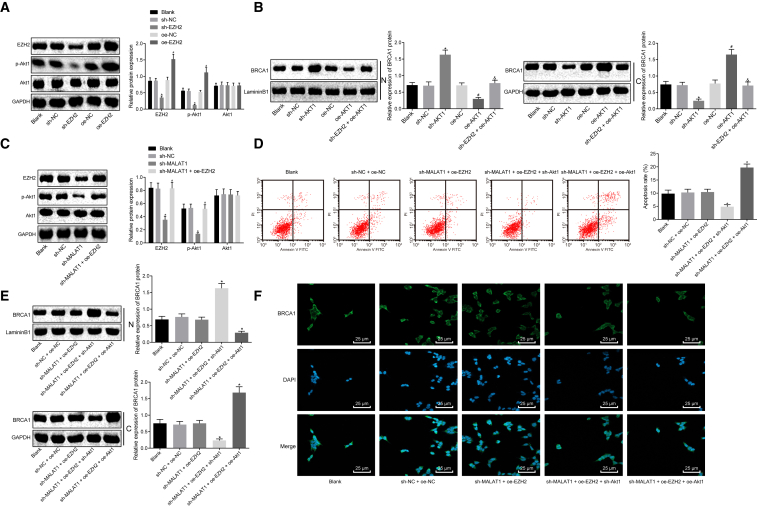
lncRNA MALAT1 Promotes the Extent of AKT-1 Phosphorylation by Recruiting EZH2 to Regulate *BRCA1* Expression in Human Skeletal Muscle Cells of Sepsis (A) The extent of AKT-1 phosphorylation normalized to GAPDH in skeletal muscle cells of sepsis determined by western blot analysis. (B) The expression of BRCA1 in the nucleus and cytoplasm normalized to LamininB1 and GAPDH, respectively, determined by western blot analysis. (C) The extent of AKT-1 phosphorylation normalized to GAPDH determined by western blot analysis. (D) The apoptosis of skeletal muscle cells measured by flow cytometry. (E) The protein expression of BRCA1 in the nuclear and cytoplasm normalized to LamininB1 determined by western blot analysis. (F) The immunofluorescence staining of BRCA1 normalized to GAPDH (×400). *p < 0.05 versus the sh-MALAT1 + oe-EZH2 group; ^#^p < 0.05 versus the sh-MALAT1 + oe-EZH2 + sh-AKT-1 group. The statistical values were measurement data, expressed as mean ± SD, and were analyzed using one-way ANOVA, followed by Tukey’s *post hoc* test. The experiment was repeated three times independently.

Western blot analysis was performed to detect the extent of AKT-1 phosphorylation in skeletal muscle cells of sepsis. As shown in Figure 5A, the extent of AKT-1 phosphorylation increased in skeletal muscle cells transfected with oe-EZH2 whereas it decreased after transfection with sh-EZH2. Next, the subcellular localization of *BRCA1* was detected by western blot analysis, which showed that silencing AKT-1 promoted the expression of BRCA1 in the nucleus and inhibited the expression of BRCA1 in the cytoplasm. The combined treatment of sh-EZH2 and oe-AKT-1 induced inhibited expression of BRCA1 in the nucleus and promoted expression in the cytoplasm (Figure 5B). Taken together, EZH2 regulated export from the nucleus of *BRCA1* in skeletal muscle cells of sepsis via regulating the extent of AKT-1 phosphorylation. Furthermore, we speculated that lncRNA MALAT1 may be involved in EZH2-mediated phosphorylation of AKT-1, thus affecting the expression and export from the nucleus of *BRCA1*. The extent of AKT-1 phosphorylation was detected after silencing lncRNA MALAT1 in skeletal muscle cells of sepsis. Western blot analysis showed that the extent of AKT-1 phosphorylation was significantly reduced upon lncRNA MALAT1 silencing, which was rescued in response to sh-MALAT1 + oe-EZH2 (Figure 5C). Flow cytometry was used to detect apoptosis of skeletal muscle cells of sepsis, which revealed that sh-MALAT1 + oe-EZH2 + sh-AKT-1 significantly inhibited cell apoptosis as compared with sh-MALAT1 + oe-EZH2 (Figure 5D). Afterward, the subcellular localization of *BRCA1* was detected by western blot analysis. It was indicated that sh-MALAT1 + oe-EZH2 + sh-AKT-1 significantly promoted the expression of BRCA1 in the nucleus and inhibited the expression of BRCA1 in the cytoplasm when compared with sh-MALAT1 + oe-EZH2 (Figure 5E), which was confirmed by an immunofluorescence assay (Figure 5F). The above results indicated that lncRNA MALAT1 regulated apoptosis of skeletal muscle cells of sepsis as well as expression and export from the nucleus of *BRCA1* via EZH2-regulated phosphorylation of AKT-1.

### Downregulation of lncRNA MALAT1 Impedes Apoptosis of Skeletal Muscle Cells and Reduces Inflammatory Factor Levels in Septic Mice

**Figure 6:**
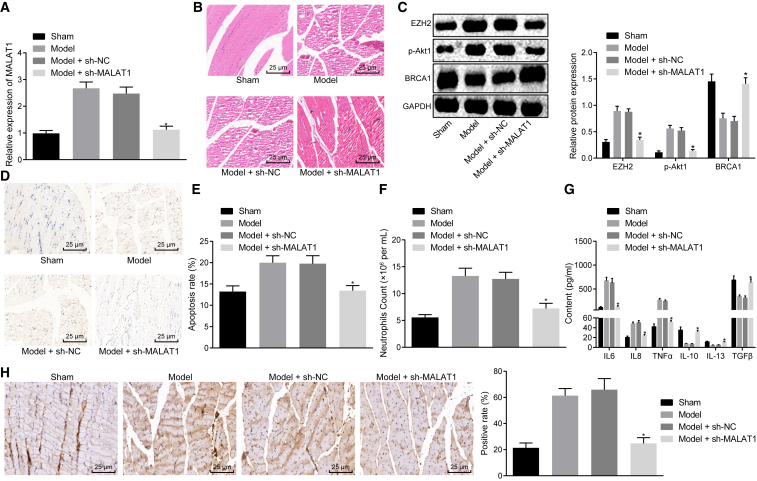
lncRNA MALAT1 Affects the Progression of Sepsis in Mice through Regulating *BRCA1* via EZH2-Regulated AKT-1 Phosphorylation *In Vivo* (A) The expression of lncRNA MALAT1 in skeletal muscle tissues of mice in response to sh-MALAT1 measured by qRT-PCR. (B) The degree of skeletal muscle injury in response to sh-MALAT1 observed by H&E staining (×400). (C) The expression of EZH2 and BRCA1 as well as the extent of AKT-1 phosphorylation normalized to GAPDH in response to sh-MALAT1 determined by western blot analysis. (D) TUNEL staining of skeletal muscle cells in response to sh-MALAT1 (×400). (E) the apoptosis rate of skeletal muscle cells measured by TUNEL staining. (F) The number of neutrophils in peripheral blood in response to sh-MALAT1 measured by flow cytometry. (G) Serum levels of IL-6, TNF-α, IL-8, IL-10, TGF-β, and IL-13 in response to sh-MALAT1 determined by ELISA. (H) Localization and expression of *BRCA1* in skeletal muscle tissues in response to sh-MALAT1 observed by immunofluorescence assay (×400); *p < 0.05 versus the sepsis + sh-NC group. The statistical values were measurement data, expressed as mean ± SD, and were analyzed using one-way ANOVA, followed by Tukey’s *post hoc* tests. N = 10. The experiment was repeated three times independently.

To validate the effect of lncRNA MALAT1 on sepsis *in vivo*, we silenced lncRNA MALAT1 in septic mice. First, qRT-PCR confirmed that lncRNA MALAT1 was successfully silenced in skeletal muscle tissues (Figure 6A). H&E staining was performed to detect the degree of skeletal muscle injury, and we found that silencing lncRNA MALAT1 could alleviate skeletal muscle injury in septic mice (Figure 6B). Subsequently, western blot analysis was used to determine the expression of EZH2 and *BRCA1* as well as the extent of AKT-1 phosphorylation in response to sh-MALAT1, which showed that the expression of EZH2 was reduced, the extent of AKT-1 phosphorylation was decreased, and the expression of *BRCA1* was increased in skeletal muscle tissues of septic mice after lncRNA MALAT1 was silenced (Figure 6C). TUNEL staining was conducted to evaluate cell apoptosis in skeletal muscle tissues. Figures 6D and 6E indicate that the number of apoptotic cells in response to sh-MALAT1 decreased significantly. Furthermore, flow cytometry showed that the number of neutrophils in peripheral blood upon sh-MALAT1 treatment decreased significantly (Figure 6F). Subsequently, the levels of inflammatory factors IL-6, TNF-α, IL-8, IL-10, TGF-β, and IL-13 in serum of septic mice were determined by ELISA. It was shown that the levels of IL-6, TNF-α, and IL-8 were reduced, and those of IL-10, TGF-β, and IL-13 were increased after silencing lncRNA MALAT1 (Figure 6G). Then, an immunofluorescence assay was performed to evaluate the subcellular localization of *BRCA1* in skeletal muscle tissues, which displayed that silencing lncRNA MALAT1 promoted the expression of *BRCA1* in the nucleus and decreased the expression of *BRCA1* in the cytoplasm in skeletal muscle tissues (Figure 6H). These results suggested that lncRNA MALAT1 affected the progression of sepsis *in vivo* through regulating *BRCA1*.

## Discussion

Sepsis represents the main cause of death in critically ill patients.^22^ In sepsis, the invading pathogen induces an immune response, which fails to return to homeostasis, thus causing a pathological syndrome, which is characterized by immune suppression and excessive inflammation.^23^ In recent years, lncRNAs have been suggested to be good candidates as biomarkers as well as therapeutic targets for sepsis.^24^ The aim of the current study was intended to investigate the underlying role of lncRNA MALAT1 in the progression of sepsis. Collectively, the key findings obtained in the investigation suggested that lncRNA MALAT1 interacting with EZH2 stimulated AKT-1 phosphorylation and decreased *BRCA1* expression and export from the nucleus, leading to the promotion of skeletal muscle cell apoptosis and levels of inflammatory factors, consequently aggravating the progression of sepsis.

qRT-PCR was initially applied to determine the expression of lncRNA MALAT1 and *BRCA1*, which revealed that lncRNA MALAT1 was highly expressed and *BRCA1* was poorly expressed in skeletal muscle tissues of septic mice. Similarly, a previous study asserted that lncRNA MALAT1 was significantly upregulated in the heart of the sepsis rat and in LPS-induced cardiac microvascular endothelial cells (CMVECs).^17^ *BRCA1* is confirmed to be a new gene in regulating metabolic function in skeletal muscle, the decline of which leads to decreased oxygen consumption and insulin signaling but increased storage of intracellular lipid.^25^ Additionally, *BRCA1* is found to be downregulated in ischemia/reperfusion (I/R) injury, and enhanced expression of *BRCA1* after I/R injury can potentially alleviate neural damage resulting from I/R via the nuclear factor erythroid-2-related factor 2-mediated antioxidant pathway.^26^ Furthermore, the expression of *BRCA1* increased significantly upon silencing lncRNA MALAT1, and, additionally, sh-MALAT1 could promote the expression of *BRCA1* in the nucleus and reduce the expression of *BRCA1* in the cytoplasm. Therefore, lncRNA MALAT1 could potentially regulate the expression and export from the nucleus of *BRCA1* in sepsis.

A prediction from the lncATLAS website and a RNA-FISH assay in the present study revealed that both lncRNA MALAT1 and EZH2 were located in the nucleus, indicating that lncRNA MALAT1 and EZH2 might interact with each other in skeletal muscle cells in sepsis. lncRNA MALAT1 has already been reported to bind with EZH2 in mantle cell lymphoma.^27^ Another study has demonstrated that MALAT1 interacts with EZH2 and that the oncogenesis induced by lncRNA MALAT1 can be inhibited by EZH2 depletion in renal cell carcinoma.^8^ There is a positive correlation between lncRNA MALAT1 and EZH2 expression in human castration-resistant prostate cancer tissues, in which the knockdown of lncRNA MALAT1 impairs EZH2 recruitment and upregulates expression of EZH2-repressed genes.^18^ In addition, downregulation of lncRNA MALAT1 and EZH2 induced by ulinastatin confers a protective effect against LPS-induced CMVEC hyperpermeability and apoptosis in sepsis.^17^ Collectively, all of the evidence illustrated that lncRNA MALAT1 functioned through recruiting EZH2 in skeletal muscle cells in sepsis.

EZH2 regulated the expression and export from the nucleus of *BRCA1* in skeletal muscle cells in sepsis via regulation of the extent of AKT-1 phosphorylation, which was indicated by the fact that silencing EZH2 promoted the expression of *BRCA1* in the nucleus and inhibited the expression of *BRCA1* in the cytoplasm, which was reversed by treatment of oe-AKT-1. According to a previous study, specific activation of AKT-1 is a contributor to EZH2-induced phenotype, and the overexpression of EZH2 induces activation of AKT-1 and *BRCA1* inhibition in breast cancer,^10^ which was consistent with our findings.

Another critical finding from the present study was that lncRNA MALAT1-mediated *BRCA1* inhibition had the capacity to stimulate the apoptosis of skeletal muscle cells as well as elevate the levels of inflammatory factors *in vivo*. lncRNA MALAT1 has been identified to be a novel inflammatory regulator in autoimmune and inflammatory diseases such as human systemic lupus erythematosus.^28^ lncRNA MALAT1 has been indicated to facilitate glucose-induced upregulation of IL-6 and TNF-α through activation of serum amyloid antigen 3 in endothelial cells.^29^ Mechanistically, lncRNA MALAT1 interacts with nuclear factor kappa-B (NF-κB) in the nucleus, consequently suppressing its DNA binding activity and then reducing the production of inflammatory factors.^30^

**Figure 7:**
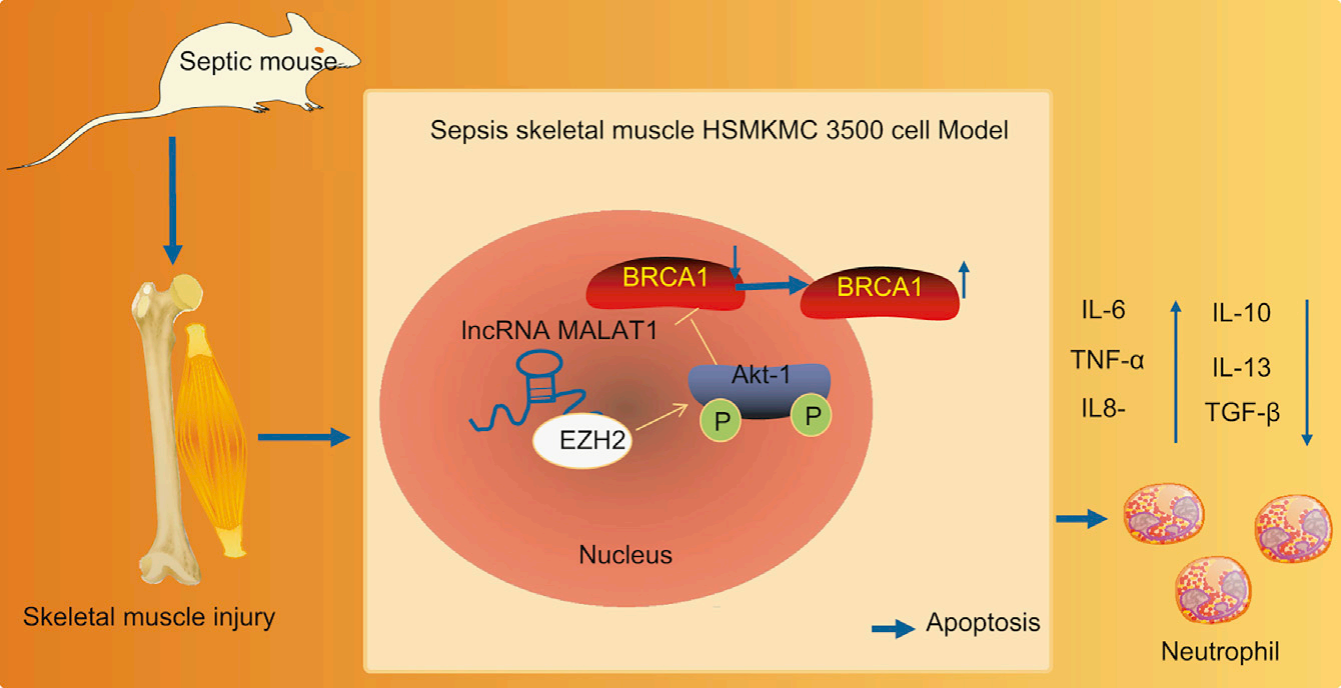
A Molecular Mechanism Map Depicting the Role of the lncRNA MALAT1/*BRCA1*/EZH2 Axis in Sepsis lncRNA MALAT1 interacting with EZH2 stimulates the phosphorylation of AKT-1 and reduces *BRCA1* expression as evidenced by elevated expression of *BRAC1* in the cytoplasm and diminished expression of *BRAC1* in the nucleus, consequently promoting apoptosis of skeletal muscle cells, increasing the expression of inflammatory factors, and aggravating the progression of sepsis.

Collectively, the expressions of lncRNA MALAT1, *BRCA1*, and EZH2 were analyzed, with the predictive results obtained indicating that lncRNA MALAT1, *BRCA1*, and EZH2 are involved in the progression of sepsis. lncRNA MALAT1 interacting with EZH2 promoted the extent of AKT-1 phosphorylation and decreased *BRCA1* expression and export from the nucleus, thus promoting skeletal muscle cell apoptosis and inflammatory responses and ultimately accelerating the progression of sepsis (Figure 7). However, further studies are still required to investigate the value of lncRNA MALAT1 in sepsis based on human skeletal muscle tissues to further validate its potential as a novel therapeutic target for sepsis treatment.

## Materials and Methods

### Ethics Statement

All animal experiment protocols were approved by the Animal Care Committee of The First Affiliated Hospital of Southwest Medical University and were in compliance with the guidelines established by the *Guide for the Care and Use of Laboratory Animals*.

### Model Establishment

A total of 100 BALB/c male mice weighing 19–22 g reared in the condition of a 12-h light/12-h dark cycle (provided by the experimental animal center of The First Affiliated Hospital of Southwest Medical University) were enrolled in this study. Then, 10 mice were randomly selected as the blank group and the sham group, respectively. The remaining 80 mice were used to establish sepsis models, and the success rate was 67.5%. The mouse model of sepsis was established as previously reported.^31^ In short, the mice were anesthetized with 3% sodium pentobarbital (no. P3761, Sigma-Aldrich, St. Louis, MO, USA) and injected intraperitoneally with 15 mg/kg LPS (bacterial endotoxin, *E. coli* LPS, serotype 0111:B4, Sigma-Aldrich, Shanghai, China). Then, the septic mice were randomly assigned into the sepsis group, the sh-NC group, and the sh-MALAT1 group, with 10 mice in each group. These mice were injected with lentivirus containing the plasmids (sh-NC or sh-MALAT1) through the tail vein. Two days later, the mice were euthanized and the skeletal muscle tissues were extracted for the following H&E staining and TUNEL staining.

### Cell Treatment

Human skeletal muscle cells (HSMKMC 3500) purchased from American Type Culture Collection (ATCC, Manassas, VA, USA) (https://www.atcc.org/) were cultured in low-glucose RPMI 1640 medium (HyClone, Logan, UT, USA) containing 10% fetal bovine serum (10100147, Gibco-BRL/Invitrogen, CA, USA) at 37°C with 5% CO_2_. Upon reaching 90% confluence, the cells were treated with 1 μg/mL LPS for 24 h and then collected. The levels of inflammatory factors TNF-α and IL-6 in the culture medium were determined. The RNA was extracted from cells and qRT-PCR was performed.

Next, oe-MALAT1, sh-MALAT1, sh-EZH2, sh-Akt-1, and sh-NC were ligated with pSIH1-H1-copGFP vector, while oe-EZH2 and oe-Akt-1 were ligated into LV5-GFP vector by GenePharma (Shanghai, China). The 293T cells were then transfected with the above plasmids by Lip2000. The cells were then cultured for 48 h and centrifuged, followed by the collection of the supernatant. Next, the titer of virus was evaluated. HSMKMC 3500 cells were infected with different virus: sh-NC, oe-NC, sh-MALAT1, oe-MALAT1, sh-EZH2, oe-EZH2, sh-Akt-1, oe-Akt-1, sh-MALAT1 + oe-NC, sh-MALAT1 + oe-EZH2, sh-MALAT1 + oe-EZH2 + sh-Akt-1, and sh-MALAT1 + oe-EZH2 + oe-Akt-1. Then, 48 h after infection, the expression efficiency of GFP was observed under a fluorescence microscope. qRT-PCR was conducted in order to detect the expression of related genes.

### qRT-PCR

Total RNA was extracted from cells and tissues with a TRIzol reagent kit (Invitrogen, Carlsbad, CA, USA). The quality and concentration of the extracted RNA were assessed using UV-visible spectrophotometry (ND-1000, NanoDrop Technologies, Wilmington, DE, USA). A total of 400 ng of RNA was reverse transcribed into cDNA based on a PrimeScript RT reagent kit (Takara Biotechnology, Dalian, China). Then, qPCR was performed according to the instructions of the SYBR Premix Ex Taq II kit (Tli RNaseH Plus; Takara Bio, Shiga, Japan) with cDNA as a template. The primers were designed by Primer Premier 5.0 software and synthesized by Guangzhou RiboBio (Guangzhou, Guangdong, China) (Table 1). Glyceraldehyde-3-phosphate dehydrogenase (GAPDH) was considered as an internal reference. The fold changes were calculated by means of relative quantification (2^−ΔΔCt^ method).^18^

**Table 1:** Primer Sequences for qRT-PCR

Gene	Sequence (5′→3′)
MALAT1 (Homo)	F: 5′-GCTCTGTGGTGTGGGATTGA-3′
	R: 5′-GTGGCAAAATGGCGGACTTT-3′
MALAT1 (Mus)	F: 5′-GAGCTCGCCAGGTTTACAGT-3′
	R: 5′-AACTACCAGCAATTCCGCCA-3′
M1	F: 5′-CCCAAGCTTGGGGTAAAGGACT GGGGCCCCGCAACT-3′
	R: 5′-TATGAAGACTTAGAAGAGTACC GCTCGAGCGG-3′
M2	F: 5′-CCCAAGCTTGGGGCATGAGGAA GGAAAAGATA-3′
	R: 5′-ACCACTCGCTTTCCCTGTCCGC TCGAGCGG-3′
M3	F: 5′-CCCAAGCTTGGGTGGTAAAAAT CCGTGAGGTC-3′
	R: 5′-CACTACCATATCCAAACAACCC GCTCGAGCGG-3′
M4	F: 5′-CCCAAGCTTGGGTGTGGTTCTC TTTTGGAATT-3′
	R: 5′-TGGTCCATTAAAGAGTGTTCCC GCTCGAGCGG-3′
M5	F: 5′-CCCAAGCTTGGGGATCAGGATT TGAGCGGAAG-3′
	R: 5′-TTGTTGTCTCTCCTGCCACACC GCTCGAGCGG-3′
M6	F: 5′-CCCAAGCTTGGGAGCGCTATTA TCCTAAGGTC-3′
	R: 5′-TTTAGAGCTTCTCCATTTCCGC TCGAGCGG-3′
BRCA1 (Homo)	F: 5′-CTGGCTTAGCAAGGAGCCAA-3′
	R: 5′-CTCTCACACAGGGGATCAGC-3′
BRCA1 (Mus)	F: 5′-GAGGCGTCGATCATCCAGAG-3′
	R: 5′-TCTTTCGAGGTTGGGTCTGC-3′
EZH2	F: 5′-GGAGTAGCTTCGCCTCTGAC-3′
	R: 5′-ACGCCCTCCAGAAACACAAT-3′
GAPDH (Homo)	F: 5′-TCAGCAATGCCTCCTGCAC-3′
	R: 5′-TCTGGGTGGCAGTGATGGC-3′
GAPDH (Mus)	F: 5′-TTAGCACCCCTGGCCAAGG-3′
	R: 5′-CTTACTCCTTGGAGGCCATG-3′
BRCA1	F: 5′-CTCTGCCGCTATCTCTGTGG-3′
	R: 5′-GCGGAATGAAAGGTCTTCGC-3′

Homo, *Homo sapiens*; Mus, *Mus musculus*; F, forward; R, reverse; EZH2, enhancer of zeste homolog 2; MALAT1, metastasis-associated lung adenocarcinoma transcript 1; BRCA1, breast cancer susceptibility gene 1; GAPDH, glyceraldehyde-3-phosphate dehydrogenase.

### Western Blot Analysis

Total proteins in cells were extracted using cell lysis buffer (R0010, C0481, Sigma-Aldrich, St. Louis, MO, USA), and the concentration was determined using a bicinchoninic acid (BCA) protein assay (Beyotime Biotechnology, Shanghai, China). Then 50 μg of protein was separated by 10% SDS-PAGE and transferred to a polyvinylidene fluoride membrane (Millipore, Billerica, MA, USA). The membrane was blocked with 5% skim milk for 1 h. Next, the membrane was incubated with the primary rabbit antibodies diluted in Tris-buffered saline with Tween 20 (TBST): BRCA1 (ab191042, 1:500), EZH2 (ab186006, 1:1,000), H3K27me3 (ab6002, 1:100), H3 (ab1791, 1:1,000), AKT-1 (ab179463, 1:10,000), phosphorylated AKT-1 (pAKT-1) (ab81283, 1:5,000), and GAPDH (ab181602, 1:10,000) at 4°C overnight. The above antibodies were purchased from Abcam (Cambridge, MA, USA). After three washes using TBST, the membrane was incubated with the horseradish peroxidase-labeled secondary antibody (ab6728, 1:1,000, Abcam, Cambridge, MA, USA) for 1 h at room temperature. The bands were visualized using enhanced chemiluminescence (Baoman Biotechnology, Shanghai, China). With GAPDH as an internal reference, ImageJ software was used to analyze the gray value. The relative protein content was expressed by the gray value of the corresponding protein band/that of GAPDH protein band.^32^

### ELISA

After 48 h of transfection, cells were collected in a 0.5-mL Eppendorf tube and placed on ice for 1 h with 60 μL of cell lysis buffer (Beyotime Biotechnology, Shanghai, China). The cells were then broken by a sonic oscillator and centrifuged at 4°C for 10 min at 12,000 rpm, with the supernatant collected. Subsequently, 10 μL of supernatant was taken out, the concentration of which was determined using a BCA kit (Beyotime Biotechnology, Shanghai, China). The concentrations of inflammatory factors such as IL-6, TNF-α, IL-8, IL-10, TGF-β, and IL-13 were measured at 562 nm by an ELISA kit according to the manufacturer’s instructions (Varioskan Flash; Thermo Scientific, Waltham, MA, USA).^32^

### H&E Staining

The skeletal muscle tissues of mice in each group were extracted and fixed with 10% neutral formaldehyde solution for more than 24 h. The paraffin-embedded sections were dewaxed with xylene twice (10 min/time), then treated with gradient ethanol (100% ethanol for 5 min, 90% ethanol for 2 min, 70% ethanol for 2 min), washed with distilled water for 2 min, and stained with hematoxylin for 7 min. In the next step, the sections were washed under tap water for 10 min to remove excess dye, rinsed again with distilled water, dehydrated with 95% ethanol for 5 s, and stained with eosin for 1 min. Then the sections were hydrated twice with gradient ethanol (100%, 95%, 75%, and 50%) (2 min/time), cleared twice with xylene (5 min/time), air-dried, and observed under an optical microscope after being sealed with neutral balsam in a fume cupboard.^33^

### Flow Cytometry

The apoptosis of skeletal muscle cells was measured by flow cytometer (Thermo Fisher Scientific, Waltham, MA, USA). The cells at passages 3–6 were inoculated into six-well plates and incubated in a 37°C incubator with 5% CO_2_. The culture medium was replaced once every 2 days. The cells were collected and resuspended with binding buffer containing 5 μL annexin V-fluorescein isothiocyanate (FITC). After 15 min of incubation at room temperature in the dark, the cells were centrifuged for 5 min at 800 rpm and the supernatant was discarded. Thereafter, the cells were resuspended with binding buffer and incubated with propidium iodide dye at 4°C in the dark for 5 min. Finally, cell apoptosis was detected and analyzed by a flow cytometer.^12^

### FISH Assay

The subcellular localization of MALAT1 and EZH2 in the skeletal muscle cells was identified using a FISH assay. The cells were then rinsed using PBS, fixed in 4% paraformaldehyde at room temperature for 10 min, penetrated with PBS containing 0.5% Triton X-100, and blocked with pre-hybridization solution at 37°C. The cells were then incubated with a FITC-conjugated MALAT1 probe (designed by Biosearch Technologies, Petaluma, CA, USA; purchased from Life Technologies. Grand Island, NY, USA) or EZH2 antibody (ab186006, 1:1,000, Abcam Cambridge, MA, USA) overnight at 37°C and incubated with secondary antibody (ab6728, 1:1,000, Abcam, Cambridge, MA, USA) at 42°C in dark. Finally, the cells were counterstained with DAPI and observed under a laser confocal microscope (Eclipse E800, Nikon, Tokyo, Japan).^34^

### ChIP Assay

Skeletal muscle cells in logarithmic growth phase were cross-linked in 1% formaldehyde for 10 min, which was then terminated with 125 mM glycine at room temperature for 5 min. Then, the cells were washed twice with pre-cooled PBS and centrifuged at 2,000 rpm for 5 min, after which the cells were collected and resuspended in cell lysate comprised of 150 mM NaCl, 50 mM Tris (pH 7.5), 5 mM EDTA, 0.005% Nonidet P-40, and 0.01% Triton X-100 to make the final concentration of 2 × 10^6^ cells per 200 mL. Subsequently, the cells were added with protease inhibitor mixture, centrifuged at 5,000 rpm for 5 min, resuspended with nuclear separation buffer, and lysed on ice for 10 min. Next, 200- to 1,000-bp chromatin fragments were obtained by ultrasonic digestion. The cells were centrifuged at 4°C at 14,000 × *g* for 10 min and the supernatant was collected. A total of 100 μL (DNA fragments) of supernatant of each group was mixed with 900 μL of ChIP dilution buffer containing 20 μL of 50× pseudoisocyanine and 60 μL of Protein A Agarose/Salmon Sperm DNA evenly at 4°C for 1 h, and centrifuged at 700 rpm for 1 min. Thereafter, the supernatant was collected, 20 μL of which was taken as input. In the experimental groups, the supernatant was incubated with 1 μL of rabbit polyclonal antibody against EZH2 (ab186006, 1:1,000, Abcam, Cambridge, MA, USA) or mouse monoclonal antibody against H3K27me3 (ab6002, 1:100, Abcam, Cambridge, MA, USA) separately. Then, the samples were washed with l mL of low-salt buffer, l mL of high-salt buffer, l mL of LiCl solution, and l mL of Tris-EDTA buffer solution (twice) and then precipitated. The DNA was eluted twice using 250 μL of ChIP wash buffer. Next, the de-cross-linking was performed using 20 μL of 5 M NaCl and then the DNA was recovered. Finally, qRT-PCR was performed to quantify DNA.^18^

### RIP Assay

The binding condition of MALAT1 with EZH2 was detected using a Magna RIP kit (Millipore, Billerica, MA, USA). The cells reaching 90% confluence were lysed in RIP lysis buffer, and subsequently the supernatant was collected, which was then divided into two equal parts. Next, 100 μL of cell extraction was incubated with 900 μL of RIP buffer containing the beads conjugated with EZH2 antibody (ab186006, 1:1,000, Abcam, Cambridge, MA, USA) or immunoglobulin G (IgG) at 4°C overnight. After instantaneous centrifugation, the centrifugal tube was placed on the magnetic separator and the supernatant was discarded. Afterward, the beads were washed with 500 μL of RIP Wash Buffer six times. The samples were incubated with protease K at 55°C for 30 min and shaken continuously to detach protein. TRIzol-chloroform was used to isolate the immunoprecipitated RNA, and the enrichment of MALAT1 was evaluated by qRT-PCR.^18^

### RNA Pull-Down Assay

An RNA fragment of MALAT1 was synthesized using T7 RNA polymerase (Ambion, Austin, TX, USA) *in vitro*, treated with an RNeasy Plus Mini kit (QIAGEN, Hilden, Germany), DNase I (QIAGEN, Hilden, Germany), and then purified using an RNeasy Mini kit. The purified RNA 3′ end was labeled with biotin RNA labeling mixture (Ambion, Austin, TX, USA). Then, 1 μg of labeled RNA was heated in RNA structure buffer (10 mmol/L Tris [pH 7], 0.1 mol/L KCl, and 10 mmo/L MgCl_2_) to 95°C for 2 min, incubated on ice for 3 min, and then placed at room temperature for 30 min to form a suitable secondary structure of RNA. Cells in the blank, sh-NC, and sh-MALAT1 groups were lysed with the addition of 3 μg of cell lysate (Sigma-Aldrich, St. Louis, MO, USA) at 4°C for 1 h. Then, the cell lysate was centrifuged at 12,000 × *g* at 4°C for 10 min and the supernatant was collected and then transferred to an RNase-free centrifugal tube. Then, 400 ng of biotinylated RNA was incubated with 500 μL of RIP buffer at room temperature for 1 h and then with streptavidin beads at room temperature for 1 h. Finally, the samples were washed with RIP buffer five times and boiled with 5× loading buffer at 95°C for 5 min. Western blot analysis was used to detect the eluted EZH2 protein.^18^

### Immunofluorescence Assay

The localization of *BRCA1* in cells was detected by immunofluorescence assay. Briefly, skeletal muscle cells were fixed with 4% paraformaldehyde for 20 min, rinsed by means of PBS for 10 min, blocked using 5% BSA and 5% goat serum for 60 min, and reacted with rabbit antibody against BRCA1 (ab191042, 1:500, Abcam, Cambridge, MA, USA). After that, the cells were incubated with FITC-labeled secondary antibody (ab6717, 1:1,000, Abcam, Cambridge, MA, USA) at room temperature for 60 min in the dark. The cells were stained with 1 mg/mL DAPI and sealed with a fluorescent tablet. Six visual fields were randomly selected in each group. All images were obtained using a Leica DC 500 camera on a microscope equipped with Leica DMRA2 fluorescence optics and analyzed with ImageJ software^35^.

### Statistical Analysis

All experimental data were processed using SPSS 21.0 statistical software (IBM, Armonk, NY, USA). The measurement data were expressed as mean ± SD. The comparisons of data following normal distribution and homogeneity of variance between two groups were conducted using an unpaired t test, while comparisons among multiple groups were assessed using one-way ANOVA, followed by Tukey’s *post hoc* tests. p < 0.05 was considered to be statistically significant.

## Author Contributions

H.Y., G.W., and J.C. designed the study. X.L., Y.B., and N.T. collated the data, carried out data analyses, and produced the initial draft of the manuscript. H.Y., L.L., and J.W. contributed to drafting the manuscript. All authors have read and approved the final submitted manuscript.

## Conflicts of Interest

The authors declare no competing interests.
