# lncRNA MALAT1 Accelerates Skeletal Muscle Cell Apoptosis and Inflammatory Response in Sepsis by Decreasing *BRCA1* Expression by Recruiting EZH2

**DOI:** 10.1016/j.omtn.2020.07.033

**Published:** 2020-09-01

**Authors:** Hui Yong, Gangming Wu, Jingyuan Chen, Xueru Liu, Yiping Bai, Ni Tang, Li Liu, Jicheng Wei

(Mol Ther Nucleic Acids. *19*, 97–108; March 6, 2020)

In the originally published version of this article, the flow cytometry apoptosis result in Figure 5D was accidentally misplaced. All results were rechecked, and the corrected figure has been replaced online and appears below. The authors regret this error.

**Figure 5:**
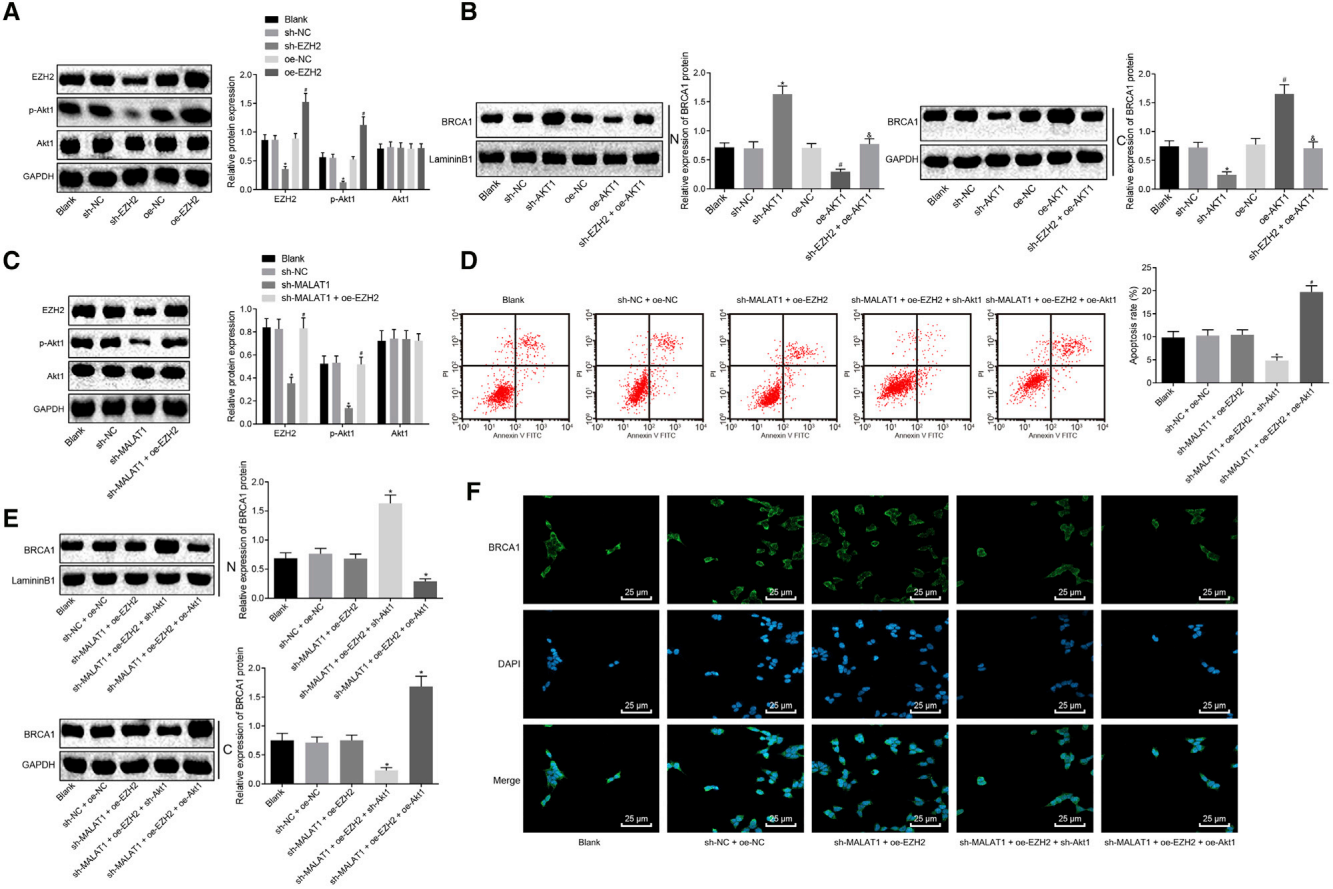
lncRNA MALAT1 Promotes the Extent of AKT-1 Phosphorylation by Recruiting EZH2 to Regulate *BRCA1* Expression in Human Skeletal Muscle Cells of Sepsis (Corrected)

**Figure 5:**
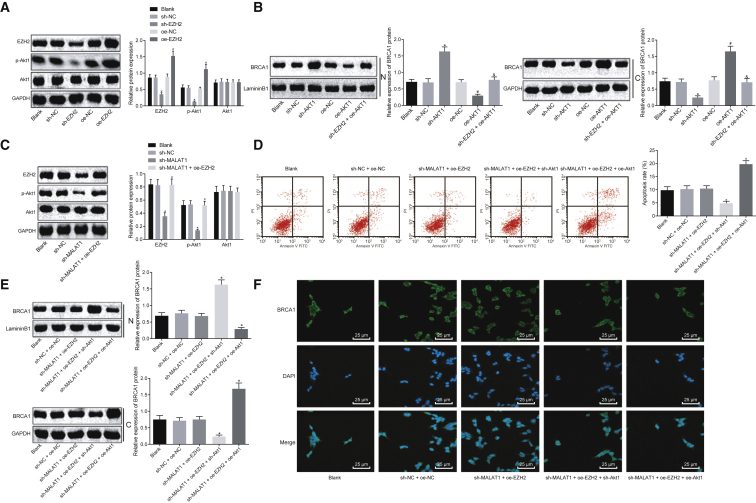
lncRNA MALAT1 Promotes the Extent of AKT-1 Phosphorylation by Recruiting EZH2 to Regulate *BRCA1* Expression in Human Skeletal Muscle Cells of Sepsis (Original)

